# Recent Advances in PLA-Based Antibacterial Food Packaging and Its Applications

**DOI:** 10.3390/molecules27185953

**Published:** 2022-09-13

**Authors:** Linying Shao, Yuewei Xi, Yunxuan Weng

**Affiliations:** 1 College of Chemistry and Materials Engineering, Beijing Technology and Business University, Beijing 100048, China; 2 Beijing Key Laboratory of Quality Evaluation Technology for Hygiene and Safety of Plastics, Beijing Technology and Business University, Beijing 100048, China

**Keywords:** food packaging, antibacterial agents, polylactic acid, biodegradable

## Abstract

In order to reduce environmental pollution and resource waste, food packaging materials should not only have good biodegradable ability but also effective antibacterial properties. Poly(lactic acid) (PLA) is the most commonly used biopolymer for food packaging applications. PLA has good physical properties, mechanical properties, biodegradability, and cell compatibility but does not have inherent antibacterial properties. Therefore, antibacterial packaging materials based on PLA need to add antibacterial agents to the polymer matrix. Natural antibacterial agents are widely used in food packaging materials due to their low toxicity. The high volatility of natural antibacterial agents restricts their application in food packaging materials. Therefore, appropriate processing methods are particularly important. This review introduces PLA-based natural antibacterial food packaging, and the composition and application of natural antibacterial agents are discussed. The properties of natural antibacterial agents, the technology of binding with the matrix, and the effect of inhibiting various bacteria are summarized.

## 1. Introduction

At present, increasingly serious environmental pollution, especially white pollution, has become a major hidden danger threatening human health. Abandoned food packaging materials are a major source of white pollution. A large number of food packaging materials are discarded and enter the soil and sea, seriously endangering human health. To change this situation, biodegradable materials have been increasingly used in the field of food packaging in recent years. As shown in Table 1 [1,2,3], Polyhydroxyalkanoates (PHAs), polylactic acid (PLA), and polycaprolactone (PCL) belong to the aliphatic polyester family and are the most commonly used biodegradable thermoplastic polymers. As a commonly used environmentally friendly polymer material, polylactic acid-based food packaging materials have become a research hotspot in recent years [4,5,6]. Polylactic acid (PLA) is a polymer material that is made of renewable resources, such as corn, cassava, sugar beet, and straw cellulose, and then polymerized into small molecular lactic acid by microbial fermentation; the degradation products are carbon dioxide and water [7,8,9]. Compared with traditional petroleum-based polymer materials, the raw materials are green and completely degradable. Compared with other degradable materials, the processing method of polylactic acid is simpler and its biocompatibility is better. Moreover, polylactic acid is non-toxic and non-irritating, which makes it suitable for use in food packaging [10].

Food packaging fulfills very important functions for the preservation of food products. According to the statistics of the Food and Agriculture Organization of the United Nations (FAO), about one-third of the food produced each year is lost or wasted due to the expiration of shelf life or deterioration caused by microbial activities. Each year, about 40–50% of fruits and vegetables, 35% of fish, 30% of grains, and 20% of dairy and meat products are consumed by microbial corrosion and the effects of oxygen and enzymes. However, for food, economic and reliable, and safe and harmless are only the basic requirements. To prolong the shelf life of food, it is also necessary to improve the antibacterial and antioxidant properties of the packaging [11]. Therefore, the development of antibacterial active packaging materials plays a key role in food safety and preservation [12,13,14,15].

Antibacterial packaging materials mainly add antibacterial agents to the polymer matrix. On the one hand, the active agents can migrate to the food, and on the other hand, the active agents can be effectively control-released, which can improve the shelf life of the food [16,17]. There are many kinds of antibacterial agents, among which natural antibacterial agents are extracted from plants so they are safe, environmentally friendly, non-toxic, and have great potential in the field of food packaging such as cloves, cinnamon, thyme, ginger, oregano, rosemary, garlic, and so on [18,19]. Therefore, the research of natural antibacterial agents has become a hot spot in the field of food packaging [20]. Due to the high volatility of natural active substances, it is necessary to choose suitable processing methods such as solvent casting, injection molding, film forming, and so on [21,22,23]. In addition, the method of coating the active agent with nanoparticles can reduce the loss of the active agent during the preparation process and can also control the release rate of the active agent [24]. When testing antibacterial performance, foodborne pathogens (such as *Escherichia coli* and *Staphylococcus aureus*) are the most selected strains [25]. These antibacterial tests can reflect the antibacterial activity of food packaging materials to some extent. Therefore, this review introduces PLA-based antibacterial food packaging and focuses on natural antibacterial packaging according to the source of the antibacterial agents. The properties of natural antibacterial agents, the technology of binding with the matrix, the effect of inhibiting various bacteria, and the effects of other related factors (such as plasticizers) are summarized.

**Table 1:** Synthesis method, degradation mechanism, characteristics, and application of polylactic acid (PLA), polyhydroxyalkanoates (PHAs), and polycaprolactone (PCL) [1,26,27,28].

	PLA	Polyhydroxyalkanoates (PHAs)	Polycaprolactone (PCL)
Synthetic method	(1) Direct polycondensation is usually accomplished by melt polycondensation, melt polycondensation solid-phase polymerization, or solution polycondensation. (2) Ring-opening polymerization of lactide.	Microbial synthesis: The structurally related or unrelated carbon sources are converted into various hydroxyacyl coenzymes As through the intrinsic carbon source metabolic pathway, and then PHAs polymerase catalyzes the polymerization of hydroxyacyl coenzyme A to synthesize PHAs.	Mainly through the ring-opening polymerization of caprolactone under the action of the catalyst.
Degradation mechanism	(1) Hydrolytic degradation: The ester bond of the main chain breaks to form carboxylic acid and alcohol, and the molecular weight decreases significantly after degradation. (2) Microbial degradation: The degrading enzymes are secreted by microorganisms and then attack the ester bond to generate cleavage, producing oligomers, dimers, and monomers.	When the carbon source is small, PHAs depolymerizing enzymes decomposed by microorganisms can degrade PHAs into oligomers and monomers for use as carbon sources and energy.	The chain segment contains ester bonds. Hydrolysis of ester bonds leads to the breaking of macromolecular chains and the decrease in the molecular weight, which is finally decomposed into carbon dioxide and water by microorganisms.
Advantage	It has a high elastic modulus, high strength, high transparency, and easy processing.	It has good biocompatibility, low cytotoxicity, and a certain gas barrier.	It has good flexibility, good impact resistance, low glass transition temperature and melting temperature, and good biocompatibility, tissue permeability, and degradation.
Shortcoming	Poor toughness, low impact strength, poor gas barrier, and high costs.	Slow crystallization speed, large crystal size, low crystal nucleus density, low mechanical strength, and high brittleness.	Low thermal stability, low modulus, strong hydrophobicity, and high costs.
Application	Medical materials such as dressings (such as hydrogels), absorbable surgical sutures, bone implants, connectors and screws for fractures, as well as in the packaging and textile industries, garden furniture manufacturing, automobile, electronics, aerospace, and other fields.	Green packaging materials, containers, electrical component shells, biomedical tissue engineering, and other fields.	Drug carriers, biomedical materials, and other fields.

## 2. Natural Antibacterial Agents

### 2.1. Classification of Natural Antibacterials

Polylactic acid (PLA) is non-toxic and non-irritating and does not have antibacterial properties. Therefore, the antibacterial properties of polylactic acid matrix composites can be improved by adding antibacterial components. In this way, they can not only retain the degradability of polylactic acid and green environmental protection but also inhibit the growth of bacteria in food and ensure food safety. Common antibacterial additives can be divided into three categories: inorganic antibacterial agents, organic antibacterial agents, and natural antibacterial agents. Because of the characteristics of natural antibacterial agents, natural antibacterial agents are selected for review in this paper.

Natural antibacterial agents refer to a class of antibacterial substances directly extracted from organisms or minerals, which can be divided into plant-derived antibacterial agents, animal-derived antibacterial agents, microbial-derived antibacterial agents, and so on [18,29,30,31], as shown in Table 2. The preparation of natural antibacterial agents not only determines its good biocompatibility, degradability, and low side effects but also limits its heat resistance and antibacterial durability so that the service life of this kind of antibacterial material is limited [31,32]. However, antibacterial agents can be loaded to solve the deactivation of antibacterial agents at high temperatures [33]. For example, natural antibacterial agents loaded with nanoparticles are used to control the release rate of active substances [34,35,36,37].

**Table 2:** Classification of natural antibacterials [18,29,30,38].

Classification	Name	Common Examples	Characteristics	Annotation
Animal-derived antibacterials	Macromolecular sugars	Chitosan, etc.	Non-toxic and non-irritating to the human body, poor heat resistance, and short duration of efficacy.	Chitosan has antibacterial activity against *Escherichia coli*, *Bacillus subtilis*, *Staphylococcus aureus,* and plant pathogens.
	Natural peptides	Antibacterial peptides, etc.		
	Amino Acids	Free amino acids, amino acid metal salt complexes, N-acylamino acids (or esters), amino acid derivatives, etc.		
Botanical antibacterials	Fruits and vegetables	Guava, etc.	The active ingredients in plants are used for sterilization, and the toxicity is low, it is not easy to produce drug resistance, and there are many kinds of drugs.	The medicinal plant resources containing antibacterial components are mainly concentrated in Compositae, Labiatae, Magnoliaceae, Aristolochia, Polygonaceae, Oleaceae, Liliaceae, Cucurbitaceae, Cyperaceae, Leguminosae, Cruciferae, and so on.
	Chinese herbal medicines	Licorice, salvia miltiorrhiza, purslane, etc.		
	Spicy spices	Cloves, ginger, garlic, cinnamon, etc.		
Microbial antibacterials	Bacteria	GM-1-1 strain, etc.	The drug resistance is strong, the type of sterilization is single, and the dosage needs to be controlled to avoid excessive abuse.	-
	Fungi	Penicillium, Aspergillus, Cladosporium, Cladosporium, etc.		
	Microbial antibiotics	-		

### 2.2. Antibacterial Mechanisms of Natural Antibacterial Agents

There are many kinds of natural antibacterial agents and their compositions are also very different; therefore, their antibacterial mechanisms are different, as shown in Table 3 [39,40,41,42,43,44].

**Table 3:** Antibacterial mechanisms of natural antibacterial agents [39,40,41,42,43,44].

Name	Antibacterial Mechanism	Annotation
Animal-derived antibacterial agents (taking chitosan as an example)	(1) The amino cations in chitosan molecules are positively charged and the cells of bacteria are negatively charged and the two interact with each other. On the one hand, they destroy the charge distribution of cell walls and cell membranes so that they cannot be synthesized normally and cells are lytic. On the other hand, a polymer membrane is formed to prevent the transport of nutrients, thus inhibiting the growth of bacteria.	The antibacterial effect of chitosan is affected by the degree of deacetylation and relative molecular weight because the degree of deacetylation is related to the number of amino groups carried by chitosan.
	(2) Chitosan molecules enter the bacteria and interact with the substances with anions in the bacteria to disrupt the normal physiological activities of the cells and achieve the bacteriostatic effect.	
Botanical antibacterial agents	(1) Destroy the cell wall or plasma membrane	There may be a variety of antibacterial mechanisms of botanical antibacterial agents, which are intertwined, interact, and influence each other.
	(2) Destroy the protein structure of the cell membrane.	
	(3) Cause the material in the cell to flow out.	
	(4) Condense the cytoplasm.	
	(5) Weaken the proton transport force	
Microbial antibacterial agents	(1) Secrete antibiotics	It plays an important role in the prevention and treatment of diseases.
	(2) Participate in the competition for nutrition and living space	
	(3) Induce the host to produce disease resistance.	
	(4) Direct action on pathogenic bacteria.	

## 3. Common Foodborne Bacteria

As shown in Table 4 [45,46,47,48,49,50], several common foodborne bacteria are listed.

**Table 4:** Common foodborne bacteria [45,46,47,48,49,50].

Name	Characteristics	Symptom
*Escherichia coli O157: H7*	Gram-staining negative, rod-shaped facultative anaerobic bacteria	The clinical symptoms are diarrhea, fever, nausea, and vomiting.
*Staphylococcus aureus*	A relatively common pathogen that is Gram-positive and is a member of the genus Staphylococcus.	When people are infected with these pathogenic bacteria, there will be gastrointestinal symptoms such as vomiting, nausea, bloody diarrhea, and so on.
*Listeria monocytogenes*	It is a Gram-positive short bacillus with 16 serotypes, 8 of which are pathogenic pathogens.	Pathogens of zoonosis

## 4. Poly(Lactic Acid)-Based Antibacterial Materials

### 4.1. Botanical Antibacterial Agent

#### 4.1.1. Curcumin

Curcumin, also known as turmeric, belongs to polyphenols with a chemical structure as shown in the figure. It is an orange-yellow water-insoluble crystal extracted from the rhizome of Zingiberaceae plants so it is a natural pigment [51]. It is soluble in organic reagents such as ethanol, methanol, and acetone and is sensitive to light, heat, alkali, and other environmental changes. Its excellent antibacterial, antioxidant, anti-inflammatory, and anti-tumor properties make it widely used in the biomedical and packaging film fields [52]. Studies have shown that the antibacterial effect of curcumin is mainly through the destruction of filamentous temperature-sensitive proteins (FtsZ protein), which is necessary for bacterial growth. Secondly, it is also related to the membrane damage caused by the combination of curcumin and peptidoglycan binding between Gram-positive and Gram-negative bacteria [53,54]. However, there are few studies on the preparation of antibacterial films by blending curcumin and PLA.

Roy et al. [55] prepared PLA-based composite films with different mass fractions of curcumin by solvent casting. The effects of the curcumin concentration on the compatibility, antibacterial properties, UV resistance, hydrophobicity, water-vapor permeability, mechanical properties, thermal stability, and oxidation resistance of the composite films were studied. As shown in the figure, compared with the control group, the pure PLA film had almost no antibacterial activity, whereas the PLA/curcumin composite film showed certain antibacterial activity and the number of bacteria decreased by 1 to 2 logarithmic cycles, which was mainly related to the antibacterial activity of curcumin [56]. Moreover, the antibacterial activity of the composite film was proportional to the concentration of curcumin, which may be related to the release rate of the curcumin in an aqueous solution.

**Figure 1:**
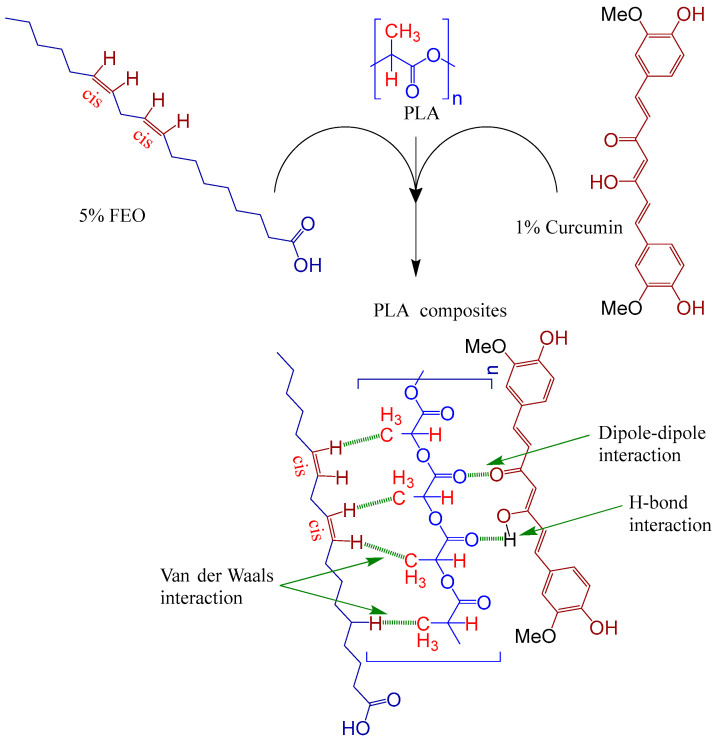
Schematic process diagram for the synthesis of the PLA-based films.

Subbuvel et al. [57] used the same method to prepare a polylactic acid film, 5 wt% polylactic acid/fenugreek essential oil (PLA/FEO^5.0%^) composite film, 1 wt% polylactic acid/curcumin (PLA/Cur^1.0%^) composite film, and PLA/Cur^1.0%^/FEO^5.0%^ composite film, as shown in Figure 1. The antibacterial activity of two foodborne pathogens, *Escherichia coli* and *Staphylococcus aureus*, was evaluated by the living bacteria counting method. The results showed that after 12 h of culture, the cell activity of *Escherichia coli* and *Staphylococcus aureus* was 3.7 log CFU/mL and 2 log CFU/mL, respectively, indicating that the composite film containing FEO was more effective in inhibiting *Staphylococcus aureus*. This may be due to the existence of the outer membrane of *Escherichia coli*, which avoids the diffusion of hydrophobic substances on the plasma membrane to some extent. In addition, some studies have shown that FEO has antibacterial activity because it contains polyphenol groups, which can interfere with the bacterial exopeptidase lipopolysaccharide layer and lead to cell degeneration [58,59,60].

**Figure 2:**
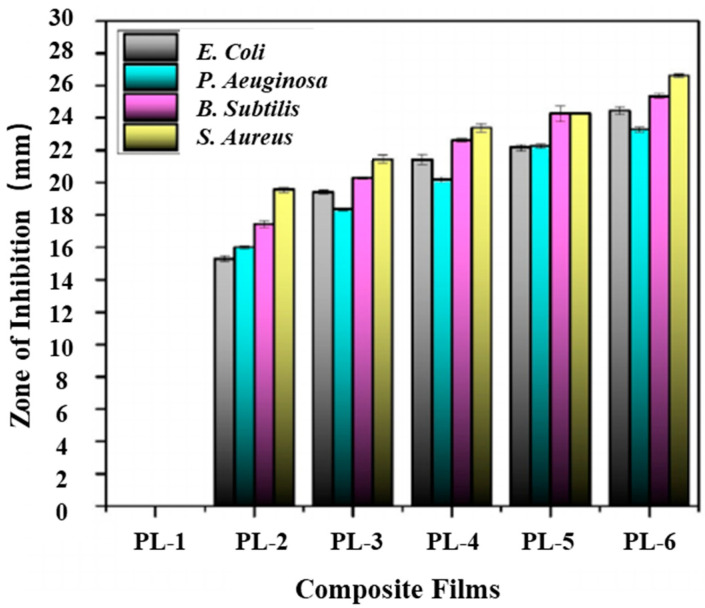
Antibacterial activity of NO- and curcumin-added various composite films. NO: neem oil Reprinted with permission from Ref. [61], published by John Wiley and Sons, 2021.

Subbuvel et al. [61] used neem oil (NO), curcumin (Cur), and polylactic acid (PLA) and the above method to prepare PLA/NO composite films with a mass fraction of 5 wt% and 10 wt% and PLA/NO/Cur composite films with an NO ratio of 10 wt% and Cur percentage of 1 wt%, 2 wt%, and 3 wt%, respectively (Table 5). The Agar diffusion method was used to detect the inhibitory effect of the sample on four kinds of bacteria: *Escherichia coli*, *Staphylococcus aureus*, *Pseudomonas aeruginosa,* and *Bacillus subskills*. It can be seen in Figure 2 that pure PLA had no inhibitory effect on any bacteria, the film with NO had good antibacterial activity against these four kinds of bacteria, and the film with NO and curcumin had a significant antibacterial effect. The antibacterial activity of NO is related to compounds such as nimbidin, nimbolin, and mahmoodi [62].

#### 4.1.2. Capsicum Oleoresin

Techawinyutham et al. [63] started with capsicum oleoresin (CO), impregnated nano-porous silica (SiCO) with CO with mass fractions of 0.5 wt%, 1 wt%, and 2 wt%, and then prepared SiCO/PLA composite films with different mass fractions using the melt-mixing method. The compositions of the samples are shown in Table 5. In this antibacterial experiment (Table 6), the antibacterial zone of each composite was determined by Duncan’s multiple range test, and it was found that the antibacterial zone of the pure PLA film was zero and had no antibacterial activity. The data show that the composite film containing 2 wt% CO had the highest antibacterial zone against *E. coli ATCC25822,4,5,12:i:- (human) US clone*, *S. Enterica Typhimurium U302 (DT104b),* and *S. aureus*. On the contrary, the concentration of CO had no significant effect on the activity of *B. subtilis* and *B. cereus*. On the one hand, the results showed that the antibacterial activity of SiCO was not lost in the processing of the composites. On the other hand, they showed that the higher the concentration of CO, the higher the surface dispersion of SiCO in the composites, which increased the contact area with microorganisms, thus enhancing the antibacterial activity of the composites. The results showed that the PLA/SiCO composite film is an excellent material for antibacterial food packaging.

In addition, Techawinyutham et al. [65] also tested the antibacterial activity of the composite films before and after accelerated weathering, as shown in Table 7. After 520 h of weathering, the bacteriostatic zone of the composite film containing 1.54 wt% SiCO was zero and could not inhibit *E. coli*. However, there was no significant change in the bacteriostatic effect of all the samples after weathering for 260 h. It can be seen in the table that because the CO in the porous silica was protected by ultraviolet light, temperature, and humidity, the antibacterial activity was not greatly affected. Therefore, the PLA composites containing SiCO showed certain antibacterial effects in different stages of weathering experiments.

#### 4.1.3. Cinnamaldehyde

**Figure 3:**
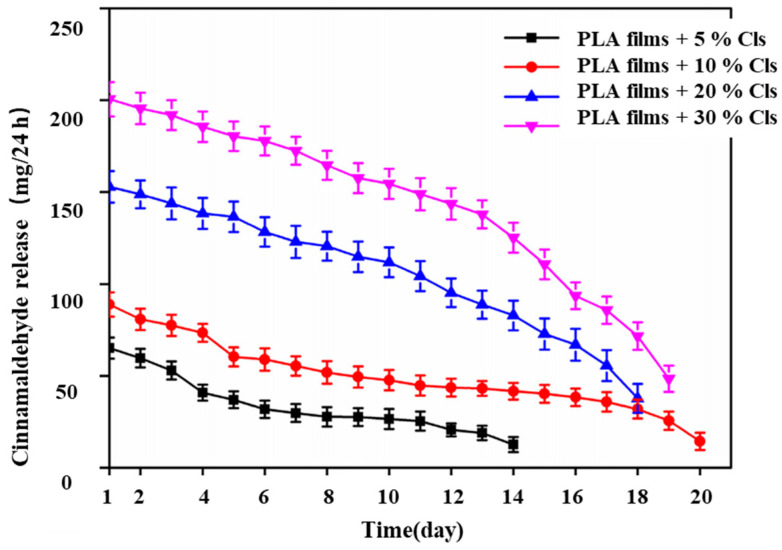
Release curves of antibacterial PLA antibacterial films with 5, 10, 20, and 30% of Cis. Reprinted with permission from Ref. [66], published by Elsevier, 2022.

To achieve the long-lasting antibacterial effect of the film, Zhang et al. [66] wrapped cinnamaldehyde (CA) with cationic-cyclodextrin using the package method and the complex was called Cls. Composite films with 0%, 5%, 10%, 20%, and 30% Cls mass fractions were produced using automatic coating equipment, in which the plasticizer tributyl citrate (ATBC) was added. The antibacterial activities of several composite films against the foodborne pathogens *Escherichia coli* and *Listeria monocytogenes* were measured (Table 8). The results showed that the inhibition rate of the 5% composite film on *Listeria monocytogenes* was as high as 60.6%, which was much higher than on *Escherichia coli* [65,67]. The other contents of the composite film showed a 100% inhibition rate on both foodborne pathogens. Some studies have shown that the antibacterial mechanism of cinnamaldehyde is that it can destroy the cell membrane of bacteria, make some substances in bacteria flow out of cells, and eventually lead to cell death [68]. In addition, the antibacterial effect of the antibacterial film is also related to the release of cinnamaldehyde in vitro, as shown in Figure 3. It can be seen in the picture that when the content of cinnamaldehyde was low (5%, 10%), the release of CA was smooth and slow and the effect was better. However, compared with the composite film containing 5% Cls, the release period of the composite film containing 10% Cls was 20 days, which was prolonged by 6 days. In a word, the composite film with 10% Cls not only produced a good antibacterial effect but also improved the morphology, crystallinity, oxygen resistance, water resistance, and tensile strength of the corresponding antibacterial PLA film. Therefore, it is suitable for fruit packaging materials.

Cui et al. [69] used carbon nanotubes (CNTs) to load cinnamaldehyde (CIN), mixed them with polylactic acid, evaporated them to make films, and finally made PLA, PLA/CNTs, PLA/CIN, and PLA/CNTs/CIN films. The results showed that the plasticizing effect of cinnamaldehyde improved the flexibility of the composite film and the layered structure of the carbon nanotubes better resisted ultraviolet radiation and controlled the release rate of cinnamaldehyde. The controlled release of cinnamaldehyde was mainly divided into two steps: first, cinnamaldehyde is released from the composite membrane to the outside of the membrane, and then further released to the surrounding environment. From the experimental data, the release of CIN was controlled by carbon nanotubes and polylactic acid double carriers so its bacteriostatic time was prolonged. On the 14th day of the experiment, it was found that there was no significant difference in the bacterial count between the PLA/CIN composite film and the PLA film. On the 21st day, it was found that the bacterial count of the PLA/CNTs/CIN composite film was the same as that of the PLA film. Therefore, PLA/CNTs/CIN films can be used in the packaging of perishable food.

**Figure 4:**
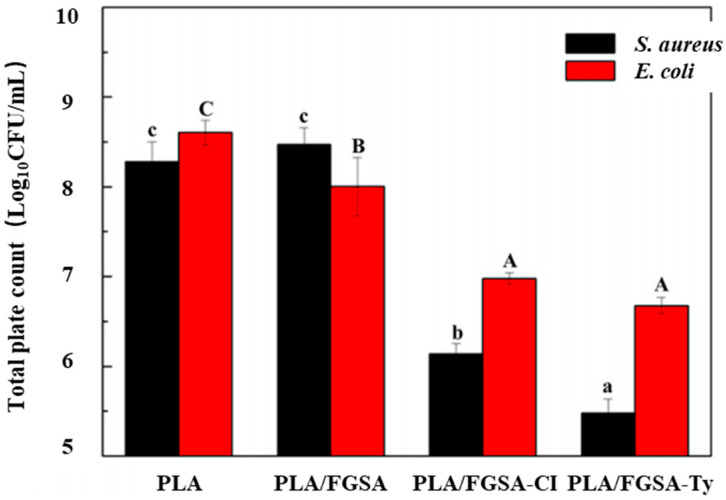
The bacteria populations in different groups were studied and shown at the end of storage. Ref. [70], published by MDPI, 2021. a–c and A–C indicate that the means are significantly different (*p* < 0.05). A value of *p* < 0.05 was considered significant.

Chen et al. [70] developed an active biodegradable bilayer film to test its antibacterial activity, antioxidation, surface morphology, and release rate of active substances and to evaluate its potential as a food packaging film. Among them, the active material release layer was a natural edible biopolymer tilapia gelatin–sodium alginate (FGSA) containing β-cyclodextrin-cinnamaldehyde (β-CD-Cl) or β-cyclodextrin-thymol (β-CD-Ty), the waterproof layer was the polylactic acid layer, and the three types of composite films were PLA/FGSA, PLA/FGSA-CI, and PLA/FGSA-Ty. The drug loadings of the inclusion complexes were 11.57% and 9.65%, respectively. As can be seen in Figure 4, the composite film containing active compounds had an inhibitory effect on *Staphylococcus aureus* and *Escherichia coli*, but the biodegradable film containing thymol had a stronger inhibitory effect. This may be related to the release efficiency of thymol in the composite film, and the effective release of active compounds can inhibit microbial growth [71,72]. In addition, in the liquid food simulation solution, except for 3% acetic acid, the release efficiency of thymol was higher than that of cinnamaldehyde, indicating that acidic liquid food is suitable for active biodegradable bilayer membranes containing cinnamaldehyde.

#### 4.1.4. Thymol and Eugenol

In order to prolong the storage time of fresh food, Ramos et al. [73] developed biodegradable active bio packaging to improve the release time of the active agents. The bioactive membrane was made of polylactic acid (PLA) as the matrix material, thymol (T) as an active agent (the content of thymol (T) was 8 wt%), and montmorillonite D43B was used as the nano-reinforcement (the contents were 2.5 wt% and 5 wt%, respectively). It was found that the addition of montmorillonite changed the diffusion path of thymol in the polylactic acid matrix and realized the controlled release of the active agent. They tested the inhibitory effect of nanocomposite films on *Escherichia coli* and *Staphylococcus aureus* using the direct contact method. The results showed that the polylactic acid-based composite film with the addition of only montmorillonite had a certain antibacterial effect, which may be related to the existence of quaternary ammonium salt in montmorillonite, which can destroy the cell activity by destroying the bacterial cell wall [74,75]. The nanocomposite film with the ternary system of thymol and montmorillonite had the highest antibacterial activity. From the point of view of practical application, there is great potential for the development of new biodegradable commercial packaging films with the addition of 8 wt% thymol and 2.5 wt% D43B to the PLA matrix.

In food packaging, a biofilm is formed on the surface of the package during transportation or storage, which provides a better living environment for microorganisms and accelerates the decay of food [76,77]. Pleva et al. [78] prepared thymol (T) and eugenol (E) films with a concentration of 3% w/w. The polymer substrates were polylactic acid (PLA), poly (adipic acid succinate) (PBAT), and poly (succinic acid succinate) (PBS). The mixed films were named PLA/ T, PLA/E, PBAT/T, PBAT/E, PBS/T, and PBS/E. The biofilm formation on the surface of the composite film was investigated using the Christensen method, a 3-(4,5-dimethylthiazol-2-yl)-2,5-diphenyltetrazolium bromide (MTT) assay, and fluorescence microscopy (Table 9). The results showed that only fluorescence microscopy could detect the biofilm on the surface of the PLA and the number of bacteria of *Stenotrophomonas maltophilia* was the least. The biofilm of PBS could be detected using the MTT method, which proved that all the tested strains could form weak biofilm on PBS. Similarly, the Christensen method showed that there was a similar situation on PBAT thin films. Pleva et al. also tested the bacteriostatic zone of each composite film and found that all the isolated bacteria from dairy products had a complete inhibitory effect except *Escherichia coli* (Table 10). In summary, these two experiments confirmed that the biodegradable polymer films with eugenol or thymol can inhibit the bacteria isolated from dairy products.

In daily life, blackberries and raspberries are very perishable and not easy to preserve, so Velázquez-Contreras et al. [79] studied the packaging products of blackberries and raspberries. They coated β-cyclodextrin (β-CDs) with a concentration of 2.5 wt% and 5 wt% thymol (β-CD-thymol) or carvacrol (β-CD-carvacrol) to prevent the volatilization of the active agent, then mixed it with polylactic acid and extruded it into pellets, which were then processed by injection molding to make packaging boxes. In the experiment on the antibacterial properties, *aerobic mesophilic bacteria* (AMB), *total coliform bacteria* (TC), *yeast,* and *mold* (YM) were selected to study the deterioration degrees of blackberries and raspberries in different periods. It was found that the package containing an active agent had an obvious inhibitory effect on the growth of yeast and mold. After 10 days of culture, the effect was more significant and the growth of yeast and mold was completely inhibited. In addition, the researchers also evaluated the flavor, smell, color, and texture of blackberries and raspberries during preservation, which showed that thymol and carvacrol delayed the physiological processes of fruit and reduced the loss of quality. Therefore, the packaging of polylactic acid-containing β-CD-thymol or β-CD-carvacrol is consistent with the principles of the bio-economy, that is, it is healthy and harmless. It has great potential in the packaging market in the future; however, more research needs to be done on the durability of this packaging.

#### 4.1.5. Mediterranean Propolis and *Thymus vulgaris* Essential Oil

Ardjoum et al. [80] studied the antibacterial activity of Mediterranean propolis (EEP) and *Thymus vulgaris* essential oil (TV-EOs) and added them to polylactic acid in different proportions to prepare bioactive antibacterial films. First of all, Ardjoum et al. used the agar disk diffusion method to determine the antibacterial activity of EEP against bacteria and fungi. EEP showed the best inhibitory effect on *Staphylococcus aureus* and *Penicillium*, and the diameters of the bacteriostatic zones were 12.1 mm and 11.58 mm, respectively. It has been reported that the antibacterial activity of propolis extract is related to its flavonoids and phenolic compounds [81,82,83]. Secondly, for the antibacterial effect of the composite film, it can be seen that the polylactic acid antibacterial film containing two active additives had an inhibitory effect on *Candida albicans* and *Escherichia coli*, and the antibacterial film containing only 10 wt% EEP and TV-Eos had a significant antibacterial effect on *Candida albicans*. In addition, the addition of two active substances had a certain plasticizing effect and improved the stability of the film. Therefore, the combination of polylactic acid film with EEP and TV-Eos is more suitable for the packaging application of active food [84].

#### 4.1.6. Clove Essential Oil

**Figure 5:**
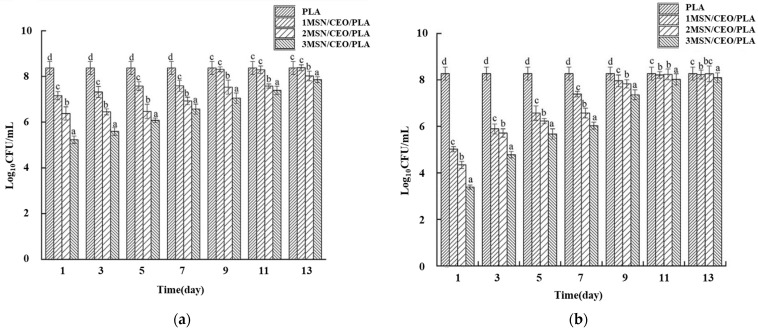
Antibacterial properties of 1MSN/CEO/PLA, 2MSN/CEO/PLA, 3MSN/CEO/PLA, and PLA films: (**a**) *E. coli*, (**b**) *S. aureus*. Reprinted with permission from Ref. [85], published by Elsevier, 2022. a–d values indicate values which were significantly different (*p* < 0.05), where a was the lowest value.

Lu et al. [85] used clove essential oil (CEO) as an antibacterial agent and PCL as the plasticizer. To improve its antibacterial aging, mesoporous silica nanoparticles were encapsulated (MSN) to control the release efficiency of clove essential oil, and then the encapsulated nanoparticles were blended with PLA and PCL to prepare composite films. The results showed that the MSN/PLA composite film had the phenomenon of agglomeration but with the addition of CEO, the agglomeration phenomenon disappeared and micropores appeared on the film surface, which may have been caused by the volatilization of CEO. Lu et al. tested the antibacterial activity of MSN/CEO/PLA composite films with different contents and different times against *Escherichia coli* and *Staphylococcus aureus* and found that the higher the content of MSN/CEO, the better the antibacterial effect. Moreover, because *Staphylococcus aureus* contains only one cell membrane outside the cell, the composite film had a stronger inhibitory effect on it. It can be seen in Figure 5 that the antibacterial activity of the composite film decreased gradually after the seventh day, which may have been related to the controlled release of CEO by the MSN carrier. In a word, the antibacterial activity of the composite film is mainly related to CEO, and the phenolic compounds in CEO can react with the bacterial cell membrane, enhance the permeability of the cell membrane, cause the loss of intracellular substances, and thus inhibit bacterial growth [86,87,88]. Therefore, MSN/CEO/PLA active composite film has wide application prospects in antibacterial food packaging materials.

**Figure 6:**
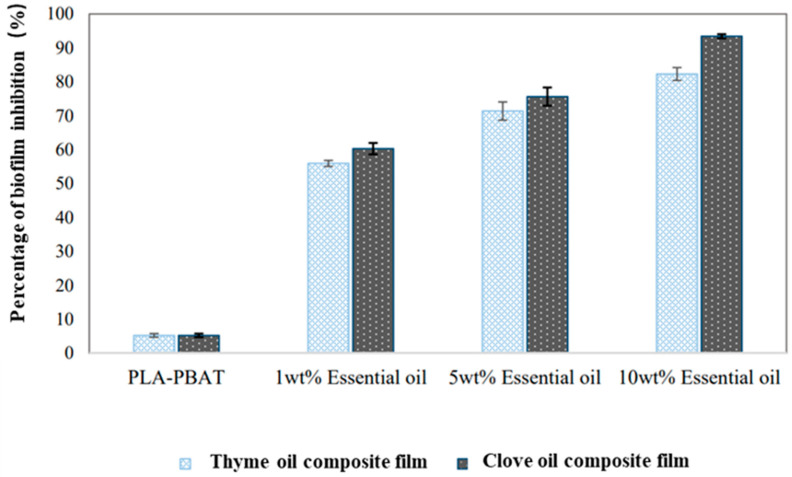
Biofilm inhibition of PLA/PBAT-thyme oil composite film and PLA/PBAT-clove oil composite film against *E. coli.* Ref. [89], published by MDPI, 2020.

Sharma et al. [89] through solvent casting, added thyme oil and clove oil of different concentrations (1 wt%, 5 wt%, and 10 wt%) to polylactic acid (PLA)-poly (butylene adipate)-terephthalate (PBAT) films to change the antibacterial properties of biodegradable films. In this study, they tested the inhibitory effects of the composite films on *Escherichia coli* and *Staphylococcus aureus* and found that clove oil had the best antibacterial activity among the two essential oils. For *Staphylococcus aureus*, when 10 wt% clove oil was added, the growth rate was 0 log CFU/mL after 24 h, whereas the antibacterial activity of the 10 wt% thyme oil composite film against *Staphylococcus aureus* was 8 h. For *Escherichia coli*, when 10 wt% clove oil was added, the growth rate was 4.4 log CFU/mL after 24 h, whereas the antibacterial activity of the 10 wt% thyme oil composite film against *E. coli* was 12 h. In addition, Sharma et al. also found that to apply the essential oil composite film to food packaging, it was necessary to test the bacterial adhesion of the composite film, which can be characterized by biofilm (*Escherichia coli*). As shown in Figure 6, the inhibition rate of the biofilm was proportional to the concentration of essential oil and the conclusion was similar to that of the antibacterial properties. The composite film with a concentration of 10 wt% of essential oil (thyme oil or clove oil) inhibited the formation of biofilm, but the inhibition rate of the clove oil composite film (93.43%) was higher than that of the thyme oil composite film (82.30%). Therefore, compared with thyme oil, the antibacterial properties and biofilm inhibition abilities of the composite films were improved after adding clove oil, which avoided the adhesion and growth of pathogens to a certain extent and improved the shelf life of packaged food. Therefore, it has a great application prospect in the field of active packaging.

Ahmed et al. [90] used poly (caprolactone) (PCL) and polyethylene glycol (PEG) as plasticizers to prepare films by blending with polylactic acid (PLA). To improve the antibacterial activity, different proportions of ZnO nanoparticles and clove essential oil (CEO) were added. In the test of the antibacterial properties of *Staphylococcus aureus* and *Escherichia coli* in vitro, it was found that *Staphylococcus aureus* decreased by 11.5 log after 1 day. However, the number of *Escherichia coli* bacteria in the PLA/PEG/PCL/CEO composite film decreased by 6 log and the PLA/PEG/PCL/ZnO/ CEO film showed a complete inhibitory effect on *Escherichia coli*. They thought that this was due to the synergism between the eugenol and ZnO nanoparticles in the clove essential oil destroying bacterial cell walls and bacterial lysis. In addition, the researchers also tested the antibacterial activity of the composite film on scrambled egg packaging and obtained a similar conclusion. Adding CEO to the PLA/PEG/PCL/ZnO composite film further enhanced the inhibitory effect of the composite film (under the same storage conditions, after 21 days, the bacteriostatic amount of *Escherichia coli* was 0 log CFU/g and *Staphylococcus aureus* decreased to 2.36 log CFU/g).

**Figure 7:**
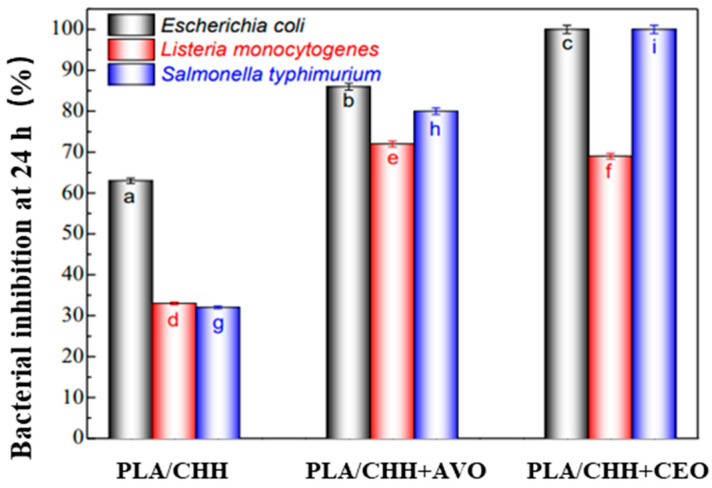
Variation of bacteria percentage inhibition determined by the CHH and oil-loaded CHH coatings. Ref. [91], published by MDPI, 2021. a–i values indicate significant differences at *p* < 0.05.

Stoleru et al. [91] used a chitosan (CHH) emulsion to encapsulate clove essential oil (CEO) and argan vegetable oil (AVO) and then fixed the oil-loaded chitosan emulsion on the polylactic acid substrate. They observed the inhibitory effect of the composite film on the growth of *Escherichia coli*, *Listeria monocytogenes,* and *Salmonella typhimurium*, as shown in Figure 7. It was found that the PLA/CHH, PLA/CHH + AVO, and PLA/CHH + CEO composite films inhibited their growth and that PLA/CHH + CEO had the best inhibitory effect. PLA/CHH + CEO had an obvious antibacterial effect on *Salmonella typhimurium* and *Escherichia coli*, and the inhibition of PLA/CHH + AVO on *Escherichia coli* was higher than that of the other two strains. Stoleru et al. also tested the antibacterial properties of three composite films used in the packaging of meat and cheese products. The results showed that the effect of polylactic acid film containing argan vegetal oil on bacterial growth on the surface of cheese was slightly greater than that of clove essential oil, whereas the effect on meat was the opposite. They believed that the antibacterial effect of the sample was due to the synergistic antibacterial effect of chitosan and the two essential oils. Finally, after 48 h of sensory analysis, it was shown that the mobility of the two essential oils in food was not obvious and they can be used in bioactive food packaging materials.

#### 4.1.7. Lignin

**Figure 8:**
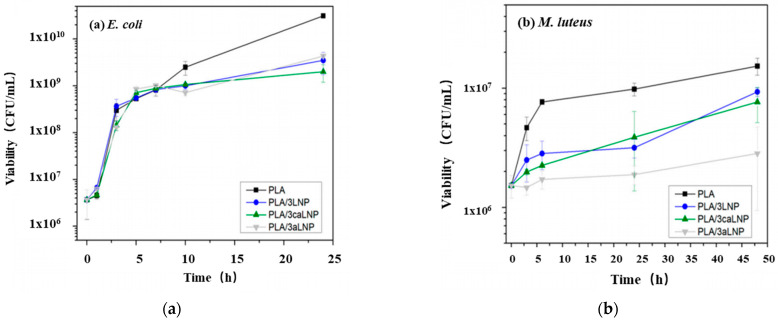
Antibacterial activity tests for PLA and PLA nanocomposite films containing 3 wt% of fillers (**a**) against *Escherichia coli*; (**b**) against *Micrococcus luteus.* Ref. [92], published by MDPI, 2020.

Cavallo et al. [92] prepared polylactic acid (PLA) films containing 1 wt% and 3 wt% lignin nanoparticles (unmodified (LNP), citric acid chemically modified (caLNP), and acetylated (aLNP)) by extrusion and determined the antibacterial activity of the composite films against *Escherichia coli* and *Micrococcus luteus*. As shown in Figure 8, the composite film containing lignin nanoparticles had a strong inhibitory effect on *Escherichia coli* after 10 h. For *Micrococcus luteus*, the slope of the composite film containing lignin nanoparticles was smaller in the exponential growth stage. In general, the composite films showed certain antibacterial activity regardless of whether lignin was modified or not. Previous studies have shown that the antibacterial mechanism of lignin is mainly the destruction of the bacterial cell wall by polyphenols, which leads to the loss of bacterial inclusions and cell lysis [93,94]. In addition, lignin will also enter the bacteria, produce monophenols, change the internal pH value of the bacteria, and inhibit the growth of bacteria [95,96,97]. Cavallo et al. also tested the migration values of the composite films and found that the migration values were all less than the required migration values. Therefore, this kind of composite film can also be used in food packaging to inhibit bacterial growth.

Luzi et al. [98] prepared a novel nanocomposite film composed of polylactic acid (PLA), lignin (LNP), zinc oxide (ZnO), and hybrid ZnO@LNP nanoparticles and applied it to the culture of adult bone marrow mesenchymal stem cells and adipose stem cells. The results showed that the stem cell proliferation rate of all the composite films was the same as that of the control group, indicating that the composite materials were non-toxic and could be used in active food packaging applications and biomedical fields.

#### 4.1.8. Grapevine Extract

**Figure 9:**
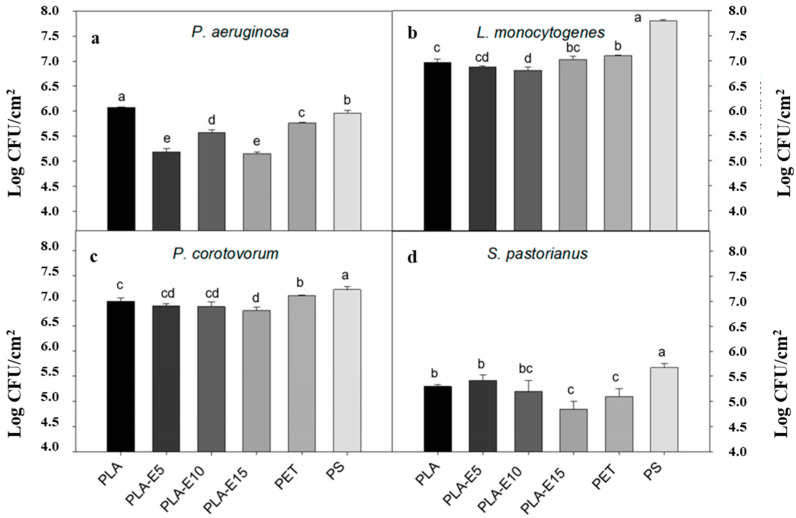
Adhesion of *P. aeruginosa* (**a**), *L. monocytogenes* (**b**), *P. corotovorum* (**c**), and *S. pastorianus* (**d**) microorganisms onto PLA, PLA-E films, polyethylene terephthalate (PET), and polystyrene (PS) samples. a–e values indicate significant differences between CFU counts according to the Duncan test (*p* < 0.05). Ref. [99], published by MDPI, 2020.

Díaz-Galindo et al. [99] used polylactic acid as a matrix, PEG as a plasticizer, grapevine extract as an antibacterial agent, and a hydraulic mechanism membrane. They tested the antibacterial effect and release efficiency of different concentrations of a grapevine extract composite film on Botrytis cinerea at different times (Table 11). The results showed that the inhibitory effect of the extract on fungi was weak and the inhibition rate was up to 35%. In addition, the released amount of grapevine extract increased with the increase in time, and the inhibition rate increased with the increase in the release amount. In addition, by observing the bacterial adhesion on the surface of the composite film (Figure 9), it was found that the bacterial adhesion from largest to smallest was *L. monocytogenes*, *P. corotovorum*, *P. aeruginosa*, and *S. pastorianus*. Except for the *L. monocytogenes* strain, the composite film with 15% grapevine extract had low adhesion to other bacteria. The addition of grapevine extract reduced the bacterial adhesion of the composite film, which may have been related to the synergism and antagonism of the phenolic compounds of the extract. In short, the polylactic acid film with grapevine extract as the active substance is suitable for the outer packaging of food that needs long-term transportation to prevent food contamination [100].

### 4.2. Animal-Derived Antibacterial Agents

#### Chitosan

Although chitosan, as an antibacterial substance, can be used in food packaging, the mechanical properties of chitosan films are poor and they are sensitive to humidity [101,102]. Therefore, in most cases, chitosan is combined with polymers to improve the properties of the composite films. Studies have shown that the antibacterial activity of chitosan is because it contains positively charged amino groups, which can bind to the negative charge on the bacterial cell membrane and cause bacterial cleavage [103,104]. Therefore, the degree of deacetylation and the molecular weight of chitosan are the key factors affecting its antibacterial properties [105].

**Figure 10:**
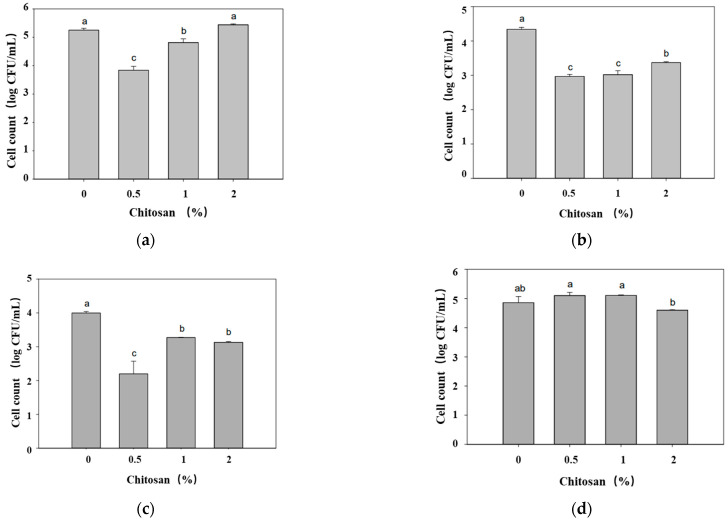
Antibacterial activity of various films of PLA and PLA containing 0.5%, 1%, and 2% of chitosan against (**a**) *Escherichia coli*; (**b**) *Staphylococcus aureus*; (**c**) *Pseudomonas fluorescens*; and (**d**) *Vibrio parahaemolyticus*. a–c values indicate significant differences at *p* < 0.05. Ref. [106], published by MDPI, 2021.

The film used by Chang et al. [106] was manufactured by the Plastics Industry Center. PLA and PBAT were mixed at 7:3 and 0%, 0.5%, 1%, and 2% chitosan powder was added, respectively, then melted and mixed, and polylactic acid film and chitosan-polylactic acid film were produced using a casting laminating machine. According to the method of ISO 22196, the antibacterial activities of all samples against *Escherichia coli*, *Staphylococcus aureus*, *Pseudomonas fluorescens,* and *Vibrio parahaemolyticus* were determined. As can be seen in Figure 10, the situation of *Vibrio parahaemolyticus* was special and all the films had poor inhibitory ability; the chitosan-polylactic acid composite film with a concentration of 2% had the strongest inhibitory ability. For *Escherichia coli* and *Staphylococcus aureus*, the antibacterial activity of the composite film was inversely proportional to the concentration of chitosan. On the whole, the antibacterial activity of the 0.5% chitosan-polylactic acid composite film was better. Some studies have shown that the antibacterial activity of chitosan is related to the degree of deacetylation, molecular weight, and pH value of the reaction. In addition, the tensile strengths (longitudinal) of the chitosan-polylactic acid composite films with 0.5%, 1% and 2% contents were about 261, 216 and 155 kgf/cm^2^, respectively, and the elongation-at-break measurements (longitudinal) were about 376%, 320% and 255%, respectively. It can be seen that the chitosan-polylactic acid composite film with 0.5% content had the highest tensile strength and elongation at break. Therefore, the 0.5% chitosan-polylactic acid composite film was selected for the fish fillet preservation experiment.

To improve the properties of polylactic acid, Singh et al. [64] used the plasticizers tributyl citrate (TEC) and glycerol triacetate (GTA) to improve the brittleness of polylactic acid, intensifier halloysite nanotubes (HNT) to improve the mechanical and thermal properties of polylactic acid, and chitosan to improve the antibacterial activity of the polylactic acid matrix. All the composite films are prepared by solvent casting with the composition as shown in Table 12. The antibacterial activity of the samples against *Escherichia coli* and *Staphylococcus aureus* was observed and was mainly characterized by antibacterial efficiency. The calculation of antibacterial efficiency (AE%) was similar to Equation (1), where B is the total number of bacteria in the pure PLA film and A is the total number of bacteria in the other composite films. It can be seen in Table 12 that the antibacterial efficiency of the chitosan composite film was significantly improved and the antibacterial efficiency of *Escherichia coli* was higher than that of *Staphylococcus aureus*. The bacteriostasis of chitosan is mainly achieved through the amino groups in the structure because there are negatively charged peptidoglycans, lipopolysaccharides, and proteins on the bacterial cell wall, which are easy to combine with amino groups, thus hindering the transport of nutrients and inhibiting bacterial growth. Hu et al. also found that the antibacterial activity of chitosan against *Escherichia coli* was higher than that of *Staphylococcus aureus*, which showed that these phenomena were caused by different structures of bacterial cell walls. There is also an outer membrane outside the cell wall of *E. coli*, whereas the cell wall of *Staphylococcus aureus* has only one layer of peptidoglycan. For *Escherichia coli*, the existence of an outer membrane increases the negative charge on the bacterial surface, thus enhancing the resistance to antibacterial substances.

(1) $$\mathrm{Antibacterial}\;\mathrm{efficiency}\;\mathrm{AE}\%=\frac{B\;-\;A}{B}\;\times \;100$$

Gomes et al. [107] extracted chitosan (Ch) from squid rings and produced three kinds of β-chitooligosaccharides (β-Cho) with different degrees of deacetylation: (1) the first kind of chitosan (β-ChoA): Mn = 1388 kDa/Mw = 186 kDa, (2) the second kind of chitosan (β-ChoB): Mn = 84 kDa/Mw = 129 kDa, and (3) the third kind of chitosan (β-ChoC): Mn = 37 kDa/Mw = 61 kDa. Then, the polylactic acid films were functionalized by plasma treatment and impregnation coating with different degrees of deacetylation of chitosan, and the antibacterial activity of the composite films against *Escherichia coli* and *Pseudomonas aeruginosa* was determined. For *Escherichia coli*, the PLA/Ch, PLA/ChoA, and PLA/ChoC biofilms showed that living cells, viable but non-culturable cells (VBNC), and culturable cells decreased by an average of 70%, 74%, and 63%, respectively (*p* < 0.001). For *Pseudomonas aeruginosa*, except for the PLA/ChoC biofilm, the number of living cells, VBNC, and culturable cells in other biofilms decreased by 73%, 52%, and 87%, respectively. In a word, the biofilm with the highest antibacterial activity was the polylactic acid film coated with natural β-chitosan (Mn = 206 kDa/Mw = 294 kDa) and depolymerized β-chitosan (ChoA, Mn = 138 kDa/Mw = 186 kDa) with the highest molecular weight.

**Figure 11:**
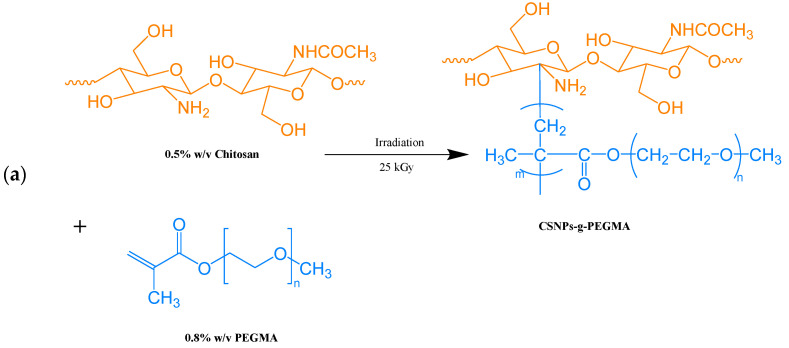

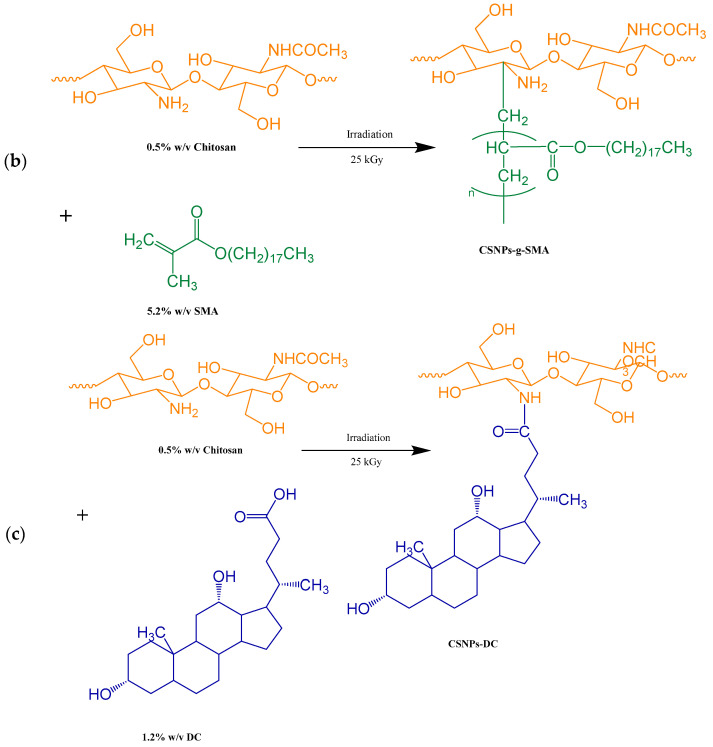
Modification reaction of CSNPs for preparing the different derivatives: (**a**) CSNPs-g-PEGMA; (**b**) CSNPs-g-PEGMA; (**c**) CSNPs-DC. Reprinted with permission from Ref. [108], published by Elsevier, 2022.

Kongkaoroptham et al. [108] prepared chitosan nanoparticles (CSNPs) by modifying chitosan with polyethylene glycol methyl methacrylate (PEGMA), stearyl methacrylate (SMA), and deoxycholic acid (DC) using radiation grafting polymerization and chemical conjugation. As shown in Figure 11, the modified CSNPs-g-pPEGMA, CSNPs-g-pSMA, and CSNPs-DC nanoparticles were blended with polylactic acid to prepare active composite films. As shown in Table 13, CSNPs-g-pSMA/PLA had the highest inhibitory ability, with bacterial activity as low as 4.67% and antibacterial activity as high as 95.29%. There was a similar phenomenon in inhibiting microorganisms in bread slices. This is because the nanoparticles still retain the amino group of chitosan after grafting and contain long-chain alkyl fatty acids, which enhance the antibacterial activity of the active composite film [109,110]. In addition, all the composite films had significant antibacterial effects on *Staphylococcus aureus*.

## 5. Conclusions and Future Developments

Food packaging materials are essential for inhibiting the growth of microorganisms in food and extending the shelf life of food so there is a need to develop safer food packaging to meet the rapidly growing needs of the industry. As a green and safe food additive, natural antibacterial agents can be applied to food packaging systems to achieve dual effects of health and environmental protection. Compared with other food preservation technologies, the use of natural antibacterial agents has the advantages of low cost and good safety, which can fundamentally inhibit the damage of microorganisms to food and ensure safety.

However, two main reasons limit the wide application of natural antibacterial agents at present: (1) the smell of natural antibacterial agents can affect the flavor of packaged food, and (2) improper processing methods can lead to the inactivation of natural antibacterial agents, declining mechanical properties of packaging materials, poor barrier performance, etc. Therefore, because of these disadvantages, the controlled release of antibacterial agents has become a hot research topic. The introduction of micro-nanostructures, such as microcapsules and nanofibers, into packaging materials helps to achieve the gradual release of antibacterial agents while maintaining the mechanical properties of the material. In addition, some natural antibacterial agents have pigments and can be used as pH chromogenic agents, such as anthocyanin and curcumin, which can be used in food packaging materials to detect the freshness of food. This has become an important research direction. In future research, molecular simulation techniques could be used to explore the release paths of natural antibacterial agents, further explore their antibacterial mechanisms, improve their preparation processes and conditions, and realize their best uses in the field of PLA-based food packaging, which can effectively inhibit bacteria, prevent corruption, and reduce environmental pollution.

## Figures and Tables

**Table 5:** Names of samples in the literature.

Reference	Films	PLA (wt%)		NO (wt%)		Curcumin (wt%)
[61]	PL-1	100		0		0
	PL-2	100		5		0
	PL-3	100		10		0
	PL-4	100		10		1
	PL-5	100		10		2
	PL-6	100		10		3
	Films	The concentration of CO in SiCO/PLA film (wt%)				
[63]	PLA	-				
	0.77 wt% SiCO	0.5				
	1.54 wt% SiCO	1				
	3.08 wt% SiCO	2				
	Films	PLA (wt%)	TEC (wt%)	GTA (wt%)	HNT (wt%)	Chitosan (wt%)
[64]	PLA	100	-	-	-	-
	PLA-TEC	90	10	-	-	-
	PLA-GTA	90	-	10	-	-
	PLA-HNT	97	-	-	3	-
	PLA-TEC-HNT	87	10	-	3	-
	PLA-GTA-HNT	87	-	10	3	-
	PLA-HNT-Chitosan	96	-	-	3	1
	PLA-TEC-HNT-Chitosan	86	10	-	3	1
	PLA-GTA-HNT-Chitosan	86	-	10	3	1

**Table 6:** Inhibition Zone [63].

Sample		Inhibition Zone (cm)						
		*B. subtilis*	*B. cereus*	*E. coli ATCC25822*	*S. enterica 4,5,12:i:- (human) US clone*	*S. enterica Enteritidis (human)*	*S. enterica Typhimurium U302 (DT104b)*	*S. aureus*
Concentration of CO (wt%)	0.5	0.28 ± 0.21	0.82 ± 0.02	0.82 ± 0.02	0.70 ± 0.04	1.08 ± 0.02	0.62 ± 0.02	0.82 ± 0.12
	1	0.28 ± 0.02	0.82 ± 0.02	0.88 ± 0.02	0.92 ± 0.02	1.05 ± 0.25	1.23 ± 0.28	0.87 ± 0.00
	2	0.30 ± 0.04	0.95 ± 0.07	0.95 ± 0.17	0.85 ± 0.07	0.98 ± 0.02	1.20 ± 0.04	1.22 ± 0.07
Concentration of CO (%) in PLA/SiCO composites	PLA	0.00 ± 0.00	0.00 ± 0.00	0.00 ± 0.00	0.00 ± 0.00	0.00 ± 0.00	0.00 ± 0.00	0.00 ± 0.00
	0.5	0.60 ± 0.00	0.60 ± 0.00	0.29 ± 0.40	0.00 ± 0.00	0.89 ± 0.12	0.60 ± 0.00	0.00 ± 0.00
	1	0.60 ± 0.00	0.62 ± 0.02	0.35 ± 0.49	0.97 ± 0.52	0.75 ± 0.21	0.87 ± 0.05	0.00 ± 0.00
	2	0.60 ± 0.00	0.60 ± 0.00	0.62 ± 0.21	1.04 ± 0.62	0.98 ± 0.07	0.97 ± 0.05	0.74 ± 0.05

**Table 7:** Inhibition zone of PLA and SiCO modified PLA composites [65].

Sample	Inhibition Zone (cm)				
	*E. coli*	*B. subtilis*	*B. cereus*	*S. enterica Typhimurium*	*S. enterica Enteritidis*
PLA	0.00 ± 0.00	0.00 ± 0.00	0.00 ± 0.00	0.00 ± 0.00	0.00 ± 0.00
0.77 wt%	0.67 ± 0.05	0.60 ± 0.00	0.60 ± 0.00	0.60 ± 0.00	0.60 ± 0.00
1.54 wt%	0.67 ± 0.02	0.60 ± 0.00	0.68 ± 0.04	0.60 ± 0.00	0.60 ± 0.00
3.08 wt%	0.60 ± 0.00	0.60 ± 0.00	0.60 ± 0.00	0.60 ± 0.00	0.60 ± 0.00
0.77 wt% 260 h	0.65 ± 0.07	0.60 ± 0.00	0.60 ± 0.00	0.60 ± 0.00	0.60 ± 0.00
1.54 wt% 260 h	0.77 ± 0.09	0.60 ± 0.00	0.60 ± 0.00	0.60 ± 0.00	0.60 ± 0.00
3.08 wt% 260 h	0.88 ± 0.00	0.60 ± 0.00	0.60 ± 0.00	0.60 ± 0.00	0.60 ± 0.00
0.77 wt% 520 h	0.60 ± 0.00	0.60 ± 0.00	0.65 ± 0.07	0.43 ± 0.07	0.57 ± 0.03
1.54 wt% 520 h	0.00 ± 0.00	0.60 ± 0.00	0.60 ± 0.00	0.57 ± 0.03	0.60 ± 0.00

**Table 8:** Antibacterial effect of PLA antibacterial films with 0, 5, 10, 20, and 30% of CIs on *Escherichia coli* and *Listeria monocytogenes* [66].

Sample Code	*E. coli*	Growth Inhibition Rate (%)	*L. monocytogenes*	Growth Inhibition Rate (%)
	10^3^		10^2^	
PLA	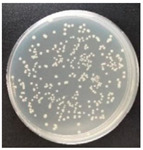	-	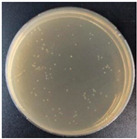	-
PLA+ 5% Cls	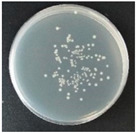	37.4	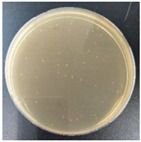	60.6
PLA+ 10% Cls	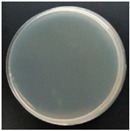	100	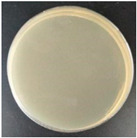	100
PLA+ 20% Cls	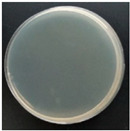	100	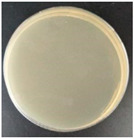	100
PLA+ 30% Cls	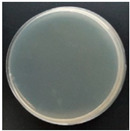	100	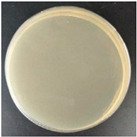	100

**Table 9:** Comparison of methods for evaluating biofilm formation for pure materials [78].

Materials	Methods	*B. tequilensis*	*B. subtilis*	*B. pumilus*	*S. maltophilia*	*E. coli*	*S. aureus*
PLA	MTT assay	−	−	−	−	−	−
	Christensen method	−	−	−	−	−	−
	Fluorescence microscopy (LIVE)	+++	+++	+++	+	+++	+++
	Fluorescence microscopy (DEAD)	+	−	−	+	++	++
PBS	MTT assay	+	+	+	+	+	+
	Christensen method	−	−	+	+	−	−
	Fluorescence microscopy (LIVE)	+	+	+	+	+	+
	Fluorescence microscopy (DEAD)	++	+	++	+	+++	+
PBAT	MTT assay	−	−	−	−	−	−
	Christensen method	+	+	+	+	+	+
	Fluorescence microscopy (LIVE)	+	+	+	+	+++	+
	Fluorescence microscopy (DEAD)	−	−	−	−	+++	−

PLA: poly(lactic) acid, PBAT: poly(butylene adipate-co-terephthalate), PBS: poly(butylene succinate). MTT assay and Christensen method: −: non-biofilm formation, +: with weak biofilm formation, ++: with strong biofilm formation (*p* < 0.003). Fluorescence microscopy: −: without microorganisms, +: 1–10 microorganisms, ++: 10–50 microorganisms, +++: >50 microorganisms.

**Table 10:** Antibacterial activity is determined using the disk diffusion method (sample 5 mm in diameter) [78].

Samples	*B. tequilensis* (mm)	*B. subtilis* (mm)	*B. pumilus* (mm)	*S. maltophilia* (mm)	*E. coli* (mm)	*S. aureus* (mm)
PLA	*	*	*	*	*	*
PLA/T	*	*	7.8 ± 1.2	12.5 ± 0.3	*	8.0 ± 0.4
PLA/E	9.5 ± 0.5	9.8 ± 0.3	7.3 ± 0.5	13.3 ± 0.3	*	9.0 ± 0.4
PBS	*	*	*	*	*	*
PBS/T	10.0 ± 0.4	9.5 ± 0.3	7.0 ± 0.4	*	10.5 ± 1.2	15.8 ± 0.5
PBS/E	11.8 ± 0.5	13.0 ± 0.7	12.3 ± 0.9	13.0 ± 0.4	15.8 ± 1.1	17.8 ± 0.5
PBAT	*	*	*	*	*	*
PBAT/T	*	7.3 ± 0.3	7.3 ± 0.3	6.3 ± 0.3	9.3 ± 0.3	10.3 ± 0.3
PBAT/E	7.8 ± 0.5	7.5 ± 0.3	9.5 ± 0.3	10.8 ± 0.3	6.3 ± 0.3	8.8 ± 0.5

PLA: poly(lactic) acid, PBAT: poly(butylene adipate-co-terephthalate), PBS: poly(butylene succinate), E: 3% *w*/*v* eugenol, T: 3% *w*/*v* thymol. *: no inhibition zone.

**Table 11:** Release of extract and inhibition of mycelial growth of *B. cinerea* onto PLA-E samples [99].

Sample	Release of Extract (mg/L)		Inhibition of Mycelial Growth (%)	
	2nd Day	7th Day	2nd Day	7th Day
PLA-E5	1.66 ± 0.12	1.90 ± 0.18	16.2 ± 2.1	4.9 ± 2.7
PLA-E10	1.73 ± 0.15	2.23 ± 0.21	22.5 ± 1.7	25.0 ± 3.9
PLA-E15	2.03 ± 0.18	4.39 ± 0.46	34.7 ± 0.4	35.8 ± 1.1

**Table 12:** Antibacterial efficiency against *E. coli* and *S. aureus*. Ref. [64], published by Elsevier, 2022.

Sample	Total Bacterial Colony		Antibacterial Efficiency (%)	
	*E. coli*	*S. aureus*	*E. coli*	*S. aureus*
PLA	32	369	-	-
PLA-HNT-Chitosan	6	108	81	71
PLA-TEC-HNT-Chitosan	7	102	78	72
PLA-GTA-HNT-Chitosan	7	102	78	72

**Table 13:** Antibacterial activities of PLA films containing CSNPs derivatives. Ref. [108], published by Elsevier, 2022.

Samples	Antibacterial Activity (CFU/mL)		Survival Degree (%)		Antibacterial Activity (%)	
	*E. coli*	*S. aureus*	*E. coli*	*S. aureus*	*E. coli*	*S. aureus*
PLA	0.07 ± 0.01	0.28 ± 0.02	85.16 ± 1.29	52.49 ± 2.98	14.84 ± 1.29	47.51 ± 2.98
CSNPs-g-PEGMA/PLA	0.20 ± 0.02	3.63 ± 0.02	63.16 ± 3.59	0.20 ± 0.00	36.84 ± 3.59	99.98 ± 0.00
CSNPs-DC/PLA	0.69 ± 0.03	1.71 ± 0.01	20.71 ± 1.06	1.93 ± 0.02	79.29 ± 3.59	98.07 ± 0.02
CSNPs-g-pSMA/PLA	1.33 ± 0.02	1.95 ± 0.00	4.67 ± 0.37	1.13 ± 0.02	95.33 ± 0.37	98.87 ± 0.02

## Data Availability

No new data were created or analyzed in this study. Data sharing is not applicable to this article.

## References

[1] Acik G. (2020). Preparation of antimicrobial and biodegradable hybrid soybean oil and poly (ʟ-lactide) based polymer with quaternized ammonium salt. Polym. Degrad. Stab..

[2] Ferreira E.F., Mouro C., Silva L., Gouveia I.C. (2022). Sustainable Packaging Material Based on PCL Nanofibers and Lavandula luisieri Essential Oil, to Preserve Museological Textiles. Polymers.

[3] Penkhrue W., Jendrossek D., Khanongnuch C., Pathom-aree W., Aizawa T., Behrens R.L., Lumyong S. (2020). Response surface method for polyhydroxybutyrate (PHB) bioplastic accumulation in Bacillus drentensis BP17 using pineapple peel. PLoS ONE.

[4] Halonen N., Pálvölgyi P.S., Bassani A., Fiorentini C., Nair R., Spigno G., Kordas K. (2020). Bio-Based Smart Materials for Food Packaging and Sensors—A Review. Front. Mater..

[5] Jeya Jeevahan J., Chandrasekaran M., Venkatesan S.P., Sriram V., Britto Joseph G., Mageshwaran G., Durairaj R.B. (2020). Scaling up difficulties and commercial aspects of edible films for food packaging: A review. Trends Food Sci. Technol..

[6] Roy S., Rhim J.-W. (2021). Anthocyanin food colorant and its application in pH-responsive color change indicator films. Crit. Rev. Food Sci. Nutr..

[7] Decorosi F., Exana M.L., Pini F., Adessi A., Messini A., Giovannetti L., Viti C. (2019). The Degradative Capabilities of New Amycolatopsis Isolates on Polylactic Acid. Microorganisms.

[8] Kliem S., Kreutzbruck M., Bonten C. (2020). Review on the Biological Degradation of Polymers in Various Environments. Materials.

[9] Drumright R.E., Gruber P.R., Henton D.E. (2000). Polylactic Acid Technology. Adv. Mater..

[10] Farah S., Anderson D.G., Langer R. (2016). Physical and mechanical properties of PLA, and their functions in widespread applications—A comprehensive review. Adv. Drug Deliv. Rev..

[11] Motelica L., Ficai D., Ficai A., Oprea O.C., Kaya D.A., Andronescu E. (2020). Biodegradable Antimicrobial Food Packaging: Trends and Perspectives. Foods.

[12] Otoni C.G., Avena-Bustillos R.J., Azeredo H.M.C., Lorevice M.V., Moura M.R., Mattoso L.H.C., McHugh T.H. (2017). Recent Advances on Edible Films Based on Fruits and Vegetables—A Review. Compr. Rev. Food Sci. Food Saf..

[13] Mukurumbira A.R., Shellie R.A., Keast R., Palombo E.A., Jadhav S.R. (2022). Encapsulation of essential oils and their application in antimicrobial active packaging. J. Food Control..

[14] Ran T., Jacqueline S., Ashraf I. (2021). Antimicrobial activity of various essential oils and their application in active packaging of frozen vegetable products. J. Food Chemistry..

[15] Xin-Jie L., Chao-Kai C., Chih-Yao H., Kuan-Chen C., Chang-Wei H. (2021). Plasma-treated polyethylene coated with polysaccharide and protein containing cinnamaldehyde for active packaging films and applications on tilapia (*Orechromis niloticus*) fillet preservation. J. Food Control..

[16] Babapour H., Jalali H., Mohammadi Nafchi A. (2021). The synergistic effects of zinc oxide nanoparticles and fennel essential oil on physicochemical, mechanical, and antibacterial properties of potato starch films. Food Sci. Nutr..

[17] Bahrami A., Delshadi R., Assadpour E., Jafari S.M., Williams L. (2020). Antimicrobial-loaded nanocarriers for food packaging applications. J. Adv. Colloid Interface Sci..

[18] El-Saber Batiha G., Hussein D.E., Algammal A.M., George T.T., Jeandet P., Al-Snafi A.E., Tiwari A., Pagnossa J.P., Lima C.M., Thorat N.D. (2021). Application of natural antimicrobials in food preservation: Recent views. Food Control.

[19] Amiri S., Moghanjougi Z.M., Bari M.R., Khaneghah A.M. (2021). Natural protective agents and their applications as bio-preservatives in the food industry. Ital. J. Food Sci..

[20] Tajkarimi M.M., Ibrahim S.A., Cliver D.O. (2010). Antimicrobial herb and spice compounds in food. Food Control.

[21] Ji M., Wu J., Sun X., Guo X., Zhu W., Li Q., Shi X., Tian Y., Wang S. (2021). Physical properties and bioactivities of fish gelatin films incorporated with cinnamaldehyde-loaded nanoemulsions and vitamin C. Lwt.

[22] Lopusiewicz L., Macieja S., Bartkowiak A., El Fray M. (2021). Antimicrobial, Antibiofilm, and Antioxidant Activity of Functional Poly(Butylene Succinate) Films Modified with Curcumin and Carvacrol. Materials.

[23] Suganthi S., Vignesh S., Kalyana Sundar J., Raj V. (2020). Fabrication of PVA polymer films with improved antibacterial activity by fine-tuning via organic acids for food packaging applications. Appl. Water Sci..

[24] Delshadi R., Bahrami A., Assadpour E., Williams L., Jafari S.M. (2021). Nano/microencapsulated natural antimicrobials to control the spoilage microorganisms and pathogens in different food products. Food Control.

[25] Cacciatore F.A., Brandelli A., Malheiros P.d.S. (2021). Combining natural antimicrobials and nanotechnology for disinfecting food surfaces and control microbial biofilm formation. Crit. Rev. Food Sci. Nutr..

[26] Niu X., Liu Y., Song Y., Han J., Pan H. (2018). Rosin modified cellulose nanofiber as a reinforcing and co-antimicrobial agents in polylactic acid/chitosan composite film for food packaging. Carbohydr. Polym..

[27] Akay O., Altinkok C., Acik G., Yuce H., Ege G.K., Genc G. (2022). Preparation of a sustainable bio-copolymer based on Luffa cylindrica cellulose and poly(varepsilon-caprolactone) for bioplastic applications. Int. J. Biol. Macromol..

[28] Agarwal A., Shaida B., Rastogi M., Singh N.B. (2022). Food Packaging Materials with Special Reference to Biopolymers-Properties and Applications. Chem. Afr..

[29] Esmaeili Y., Paidari S., Baghbaderani S.A., Nateghi L., Al-Hassan A.A., Ariffin F. (2021). Essential oils as natural antimicrobial agents in postharvest treatments of fruits and vegetables: A review. J. Food Meas. Charact..

[30] Punia Bangar S., Chaudhary V., Thakur N., Kajla P., Kumar M., Trif M. (2021). Natural Antimicrobials as Additives for Edible Food Packaging Applications: A Review. Foods.

[31] Rodrigo D., Palop A. (2021). Applications of Natural Antimicrobials in Food Packaging and Preservation. Foods.

[32] Jafarzadeh S., Nafchi A.M., Salehabadi A., Oladzad-Abbasabadi N., Jafari S.M. (2021). Application of bio-nanocomposite films and edible coatings for extending the shelf life of fresh fruits and vegetables. Adv. Colloid Interface Sci..

[33] Yuan Z., Jiejie A., Hongxia S., Bo L., Dongwu L., Chongxing H. (2022). Antimicrobial food packaging integrating polysaccharide-based substrates with green antimicrobial agents: A sustainable path. Food Res. Int..

[34] Qi G., Gengan D., Hang J., Qiuxia F., Zhouli W., Zhenpeng G., Tianli Y., Yahong Y. (2021). Essential oils encapsulated by biopolymers as antimicrobials in fruits and vegetables: A review. Food Biosci..

[35] Akbari-Alavijeh S., Shaddel R., Jafari S.M. (2020). Encapsulation of food bioactives and nutraceuticals by various chitosan-based nanocarriers. Food Hydrocoll..

[36] Bashiri S., Ghanbarzadeh B., Ayaseh A., Dehghannya J., Ehsani A. (2020). Preparation and characterization of chitosan-coated nanostructured lipid carriers (CH-NLC) containing cinnamon essential oil for enriching milk and anti-oxidant activity. LWT.

[37] Valencia G.A., Zare E.N., Makvandi P., Gutiérrez T.J. (2019). Self-Assembled Carbohydrate Polymers for Food Applications: A Review. Compr. Rev. Food Sci. Food Saf..

[38] Xiaoxian H., Yaofa L., Dandan Z., Yongguo J., Haobo J., Long S. (2022). Preparation and characterization of edible carboxymethyl cellulose films containing natural antibacterial agents: Lysozyme. Food Chem..

[39] Bae J.-Y., Seo Y.-H., Oh S.-W. (2022). Antibacterial activities of polyphenols against foodborne pathogens and their application as antibacterial agents. J. Food Sci. Biotechnol..

[40] Kennedy C.W., Macário d.O.A., Silva S.I.B.d., Guimarães S.V.B., Carvalho d.S.E.K., Oliveira A.J.V.d., Sant’Anna d.S.A.P., Menezes L.V.L.d., Santos C.M.T.d., Vanusa d.S.M. (2022). Antibacterial mechanism of Eugenia stipitata McVaugh essential oil and synergistic effect against Staphylococcus aureus. J. South Afr. J. Bot..

[41] Yong-xin L., Famous E., Man L., Kunlong Y., Weifa Z., Jun T. (2022). Antimicrobial mechanisms of spice essential oils and application in food industry. Food Chem..

[42] Yingying Z., Jinfeng W., Changqin L., Ahmed A.F., Zhenhua L., Changyang M. (2022). A comprehensive review on mechanism of natural products against Staphylococcus aureus. Future Foods.

[43] Siwei L., Xinyi H., Ruifei W., Meimei F., Yigang Y., Xinglong X. (2022). The combination of thymol and cinnamaldehyde reduces the survival and virulence of Listeria monocytogenes on autoclaved chicken breast. J. Appl. Microbiol..

[44] Yunhui B., Jian H., Ke S., Jie G., Xianwu Z., Shima L. (2022). Functionalization and Antibacterial Applications of Cellulose-Based Composite Hydrogels. Polymers.

[45] Soyul L., Areum H., Jae-Hyun Y., Sun-Young L. (2022). Growth evaluation of *Escherichia coli* O157:H7, Salmonella typhimurium, and Listeria monocytogenes in fresh fruit and vegetable juices via predictive modeling. LWT.

[46] Zhuangzhuang B., Xianbao X., Cong W., Tan W., Chuanyu S., Shuangxi L., Daoliang L. (2022). A comprehensive review of detection methods for *Escherichia coli* O157:H7. Trends Anal. Chem..

[47] Bousiakou L.G., Qindeel R., Al-Dossary O.M., Kalkani H. (2022). Synthesis and characterization of graphene oxide (GO) sheets for pathogen inhibition: *Escherichia coli*, Staphylococcus aureus and Pseudomonas aeruginosa. J. King Saud Univ.-Sci..

[48] Shamprasad B.R., Lotha R., Nagarajan S., Sivasubramanian A. (2022). Metal nanoparticles functionalized with nutraceutical Kaempferitrin from edible Crotalaria juncea, exert potent antimicrobial and antibiofilm effects against Methicillin-resistant Staphylococcus aureus. Sci. Rep..

[49] Mao Z., Hui L., Keren A.A., Yigang Y., Xinglong X. (2022). Effects of citronellal on growth and enterotoxins production in Staphylococcus aureus ATCC 29213. Toxicon Off. J. Int. Soc. Toxinol..

[50] Ereno T.L., Henrique B.T., Aparecida R.d.S.E., Arruda S.J., Koutsodontis C.-C.C., Augusto N.L., Seiti Y.R., Gonçalves P.J., Santos B.L.d. (2022). Pure and mixed biofilms formation of Listeria monocytogenes and Salmonella Typhimurium on polypropylene surfaces. LWT.

[51] Roy S., Rhim J.W. (2020). Curcumin Incorporated Poly(Butylene Adipate-co-Terephthalate) Film with Improved Water Vapor Barrier and Antioxidant Properties. Materials.

[52] Roy S., Rhim J.W. (2020). Carboxymethyl cellulose-based antioxidant and antimicrobial active packaging film incorporated with curcumin and zinc oxide. Int. J. Biol. Macromol..

[53] Kaur S., Modi N.H., Panda D., Roy N. (2010). Probing the binding site of curcumin in *Escherichia coli* and Bacillus subtilis FtsZ—A structural insight to unveil antibacterial activity of curcumin. Eur. J. Med. Chem..

[54] Rai D., Singh J.K., Roy N., Panda D. (2008). Curcumin inhibits FtsZ assembly: An attractive mechanism for its antibacterial activity. Biochem. J..

[55] Roy S., Rhim J.W. (2020). Preparation of bioactive functional poly(lactic acid)/curcumin composite film for food packaging application. Int. J. Biol. Macromol..

[56] Zorofchian M.S., Abdul K.H., Pouya H., Hassan T., Sazaly A., Keivan Z. (2014). A review on antibacterial, antiviral, and antifungal activity of curcumin. BioMed Res. Int..

[57] Subbuvel M., Kavan P. (2022). Preparation and characterization of polylactic acid/fenugreek essential oil/curcumin composite films for food packaging applications. Int. J. Biol. Macromol..

[58] Akbari S., Abdurahman N.H., Yunus R.M., Alara O.R., Abayomi O.O. (2019). Extraction, characterization and antioxidant activity of fenugreek (Trigonella-Foenum Graecum) seed oil. Mater. Sci. Energy Technol..

[59] Al-Timimi L.A.N. (2019). Antibacterial and Anticancer Activities of Fenugreek Seed Extract. Asian Pac. J. Cancer Prev..

[60] Arfat Y.A., Ahmed J., Ejaz M., Mullah M. (2018). Polylactide/graphene oxide nanosheets/clove essential oil composite films for potential food packaging applications. Int. J. Biol. Macromol..

[61] Subbuvel M., Kavan P. (2021). Development and investigation of antibacterial and antioxidant characteristics of poly lactic acid films blended with neem oil and curcumin. J. Appl. Polym. Sci..

[62] Thiyagu T.T., Rajeswari N. (2019). Effect of nanosilica and neem tree oil on antimicrobial, thermal, mechanical and electrical insulate of biodegradable composite film. Mater. Res. Express.

[63] Techawinyutham L., Siengchin S., Parameswaranpillai J., Dangtungee R. (2019). Antibacterial and thermomechanical properties of composites of polylactic acid modified with capsicum oleoresin-impregnated nanoporous silica. J. Appl. Polym. Sci..

[64] Singh A.A., Sharma S., Srivastava M., Majumdar A. (2020). Modulating the properties of polylactic acid for packaging applications using biobased plasticizers and naturally obtained fillers. Int. J. Biol. Macromol..

[65] Techawinyutham L., Siengchin S., Dangtungee R., Parameswaranpillai J. (2019). Influence of accelerated weathering on the thermo-mechanical, antibacterial, and rheological properties of polylactic acid incorporated with porous silica-containing varying amount of capsicum oleoresin. Compos. Part B Eng..

[66] Zhang L., Huang C., Xu Y., Huang H., Zhao H., Wang J., Wang S. (2020). Synthesis and characterization of antibacterial polylactic acid film incorporated with cinnamaldehyde inclusions for fruit packaging. Int. J. Biol. Macromol..

[67] Bilbao-Sainz C., Chiou B.-S., Du W.-X., Gregorsky K.S., Orts W.J. (2012). Influence of Disperse Phase Characteristics on Stability, Physical and Antimicrobial Properties of Emulsions Containing Cinnamaldehyde. J. Am. Oil Chem. Soc..

[68] Haghighi H., Biard S., Bigi F., De Leo R., Bedin E., Pfeifer F., Siesler H.W., Licciardello F., Pulvirenti A. (2019). Comprehensive characterization of active chitosan-gelatin blend films enriched with different essential oils. Food Hydrocoll..

[69] Cui R., Yan J., Cao J., Qin Y., Yuan M., Li L. (2021). Release properties of cinnamaldehyde loaded by montmorillonite in chitosan-based antibacterial food packaging. Int. J. Food Sci. Technol..

[70] Chen J., Li Y., Shi W., Zheng H., Wang L., Li L. (2021). Release of Cinnamaldehyde and Thymol from PLA/Tilapia Fish Gelatin-Sodium Alginate Bilayer Films to Liquid and Solid Food Simulants, and Japanese Sea Bass: A Comparative Study. Molecules.

[71] Bahmid N.A., Heising J., Dekker M. (2021). Multiresponse kinetic modelling of the formation, release, and degradation of allyl isothiocyanate from ground mustard seeds to improve active packaging. J. Food Eng..

[72] Benbettaïeb N., Mahfoudh R., Moundanga S., Brachais C.-H., Chambin O., Debeaufort F. (2020). Modeling of the release kinetics of phenolic acids embedded in gelatin/chitosan bioactive-packaging films: Influence of both water activity and viscosity of the food simulant on the film structure and antioxidant activity. Int. J. Biol. Macromol..

[73] Ramos M., Fortunati E., Beltran A., Peltzer M., Cristofaro F., Visai L., Valente A.J.M., Jimenez A., Kenny J.M., Garrigos M.C. (2020). Controlled Release, Disintegration, Antioxidant, and Antimicrobial Properties of Poly (Lactic Acid)/Thymol/Nanoclay Composites. Polymers.

[74] de Azeredo H.M.C. (2013). Antimicrobial nanostructures in food packaging. Trends Food Sci. Technol..

[75] Hong S.-I., Rhim J.-W. (2008). Antimicrobial Activity of Organically Modified Nano-Clays. J. Nanosci. Nanotechnol..

[76] Azeredo J., Azevedo N.F., Briandet R., Cerca N., Coenye T., Costa A.R., Desvaux M., Di Bonaventura G., Hébraud M., Jaglic Z. (2016). Critical review on biofilm methods. Crit. Rev. Microbiol..

[77] Bridier A., Sanchez-Vizuete P., Guilbaud M., Piard J.C., Naïtali M., Briandet R. (2015). Biofilm-associated persistence of food-borne pathogens. Food Microbiol..

[78] Pleva P., Bartosova L., Macalova D., Zalesakova L., Sedlarikova J., Janalikova M. (2021). Biofilm Formation Reduction by Eugenol and Thymol on Biodegradable Food Packaging Material. Foods.

[79] Velazquez-Contreras F., Garcia-Caldera N., Padilla de la Rosa J.D., Martinez-Romero D., Nunez-Delicado E., Gabaldon J.A. (2021). Effect of PLA Active Packaging Containing Monoterpene-Cyclodextrin Complexes on Berries Preservation. Polymers.

[80] Ardjoum N., Chibani N., Shankar S., Fadhel Y.B., Djidjelli H., Lacroix M. (2021). Development of antimicrobial films based on poly(lactic acid) incorporated with Thymus vulgaris essential oil and ethanolic extract of Mediterranean propolis. Int. J. Biol. Macromol..

[81] Gargouri W., Osés S.M., Fernández-Muiño M.A., Sancho M.T., Kechaou N. (2019). Evaluation of bioactive compounds and biological activities of Tunisian propolis. Lwt.

[82] Ardjoum N., Chibani N., Boukerrou A., Djidjelli H. (2021). Study of Antimicrobial Activities of Thyme and Propolis of PLA films. Macromol. Symp..

[83] Safaei M., Roosta Azad R. (2019). Preparation and characterization of poly-lactic acid based films containing propolis ethanolic extract to be used in dry meat sausage packaging. J. Food Sci. Technol..

[84] Abou N.R., Rawan M., Rim W., Dany E.O., Marc S.J., Ziad F. (2021). Beehive Products as Antibacterial Agents: A Review. Antibiotics.

[85] Lu W., Cui R., Zhu B., Qin Y., Cheng G., Li L., Yuan M. (2021). Influence of clove essential oil immobilized in mesoporous silica nanoparticles on the functional properties of poly(lactic acid) biocomposite food packaging film. J. Mater. Res. Technol..

[86] Hadidi M., Pouramin S., Adinepour F., Haghani S., Jafari S.M. (2020). Chitosan nanoparticles loaded with clove essential oil: Characterization, antioxidant and antibacterial activities. Carbohydr. Polym..

[87] Ortiz C.M., Salgado P.R., Dufresne A., Mauri A.N. (2018). Microfibrillated cellulose addition improved the physicochemical and bioactive properties of biodegradable films based on soy protein and clove essential oil. Food Hydrocoll..

[88] Song N.-B., Lee J.-H., Al Mijan M., Song K.B. (2014). Development of a chicken feather protein film containing clove oil and its application in smoked salmon packaging. LWT-Food Sci. Technol..

[89] Sharma S., Barkauskaite S., Duffy B., Jaiswal A.K., Jaiswal S. (2020). Characterization and Antimicrobial Activity of Biodegradable Active Packaging Enriched with Clove and Thyme Essential Oil for Food Packaging Application. Foods.

[90] Ahmed J., Mulla M., Jacob H., Luciano G., Bini T.B., Almusallam A. (2019). Polylactide/poly(ε-caprolactone)/zinc oxide/clove essential oil composite antimicrobial films for scrambled egg packaging. Food Packag. Shelf Life.

[91] Stoleru E., Vasile C., Irimia A., Brebu M. (2021). Towards a Bioactive Food Packaging: Poly(Lactic Acid) Surface Functionalized by Chitosan Coating Embedding Clove and Argan Oils. Molecules.

[92] Cavallo E., He X., Luzi F., Dominici F., Cerrutti P., Bernal C., Foresti M.L., Torre L., Puglia D. (2020). UV Protective, Antioxidant, Antibacterial and Compostable Polylactic Acid Composites Containing Pristine and Chemically Modified Lignin Nanoparticles. Molecules.

[93] Yang W., Fortunati E., Dominici F., Giovanale G., Mazzaglia A., Balestra G.M., Kenny J.M., Puglia D. (2016). Synergic effect of cellulose and lignin nanostructures in PLA based systems for food antibacterial packaging. Eur. Polym. J..

[94] Qin L., Li W.-C., Liu L., Zhu J.-Q., Li X., Li B.-Z., Yuan Y.-J. (2016). Inhibition of lignin-derived phenolic compounds to cellulase. Biotechnol. Biofuels.

[95] Yang W., Fortunati E., Dominici F., Giovanale G., Mazzaglia A., Balestra G.M., Kenny J.M., Puglia D. (2016). Effect of cellulose and lignin on disintegration, antimicrobial and antioxidant properties of PLA active films. Int. J. Biol. Macromol..

[96] Zemek J., Košíková B., Augustín J., Joniak D. (1979). Antibiotic properties of lignin components. Folia Microbiol..

[97] Yang W., Fortunati E., Gao D., Balestra G.M., Giovanale G., He X., Torre L., Kenny J.M., Puglia D. (2018). Valorization of Acid Isolated High Yield Lignin Nanoparticles as Innovative Antioxidant/Antimicrobial Organic Materials. ACS Sustain. Chem. Eng..

[98] Luzi F., Tortorella I., Di Michele A., Dominici F., Argentati C., Morena F., Torre L., Puglia D., Martino S. (2020). Novel Nanocomposite PLA Films with Lignin/Zinc Oxide Hybrids: Design, Characterization, Interaction with Mesenchymal Stem Cells. Nanomaterials.

[99] Diaz-Galindo E.P., Nesic A., Cabrera-Barjas G., Dublan-Garcia O., Ventura-Aguilar R.I., Vazquez-Armenta F.J., Aguilar-Montes de Oca S., Mardones C., Ayala-Zavala J.F. (2020). Physico-Chemical and Antiadhesive Properties of Poly(Lactic Acid)/Grapevine Cane Extract Films against Food Pathogenic Microorganisms. Polymers.

[100] Kim T., Kim J.-H., Oh S.-W. (2021). Grapefruit Seed Extract as a Natural Food Antimicrobial: A Review. Food Bioprocess Technol..

[101] Chang S.-H., Chen C.-H., Tsai G.-J. (2020). Effects of Chitosan on Clostridium perfringens and Application in the Preservation of Pork Sausage. Mar. Drugs.

[102] Sébastien F., Stéphane G., Copinet A., Coma V. (2006). Novel biodegradable films made from chitosan and poly(lactic acid) with antifungal properties against mycotoxinogen strains. Carbohydr. Polym..

[103] Tsai G.-J., Su W.-H. (1999). Antibacterial Activity of Shrimp Chitosan against *Escherichia coli*. J. Food Prot..

[104] Yuan G., Lv H., Tang W., Zhang X., Sun H. (2016). Effect of chitosan coating combined with pomegranate peel extract on the quality of Pacific white shrimp during iced storage. Food Control.

[105] Chang S.-H., Lin H.-T.V., Wu G.-J., Tsai G.J. (2015). pH Effects on solubility, zeta potential, and correlation between antibacterial activity and molecular weight of chitosan. Carbohydr. Polym..

[106] Chang S.H., Chen Y.J., Tseng H.J., Hsiao H.I., Chai H.J., Shang K.C., Pan C.L., Tsai G.J. (2021). Antibacterial Activity of Chitosan-Polylactate Fabricated Plastic Film and Its Application on the Preservation of Fish Fillet. Polymers.

[107] Gomes L.C., Faria S.I., Valcarcel J., Vazquez J.A., Cerqueira M.A., Pastrana L., Bourbon A.I., Mergulhao F.J. (2021). The Effect of Molecular Weight on the Antimicrobial Activity of Chitosan from Loligo opalescens for Food Packaging Applications. Mar. Drugs.

[108] Kongkaoroptham P., Piroonpan T., Pasanphan W. (2021). Chitosan nanoparticles based on their derivatives as antioxidant and antibacterial additives for active bioplastic packaging. Carbohydr. Polym..

[109] Priscilla J., Arul Dhas D., Hubert Joe I., Balachandran S. (2020). Experimental and theoretical spectroscopic analysis, hydrogen bonding, reduced density gradient and antibacterial activity study on 2-Phenyl quinoline alkaloid. Chem. Phys..

[110] Ivanova E.P., Nguyen S.H., Guo Y., Baulin V.A., Webb H.K., Truong V.K., Wandiyanto J.V., Garvey C.J., Mahon P.J., Mainwaring D.E. (2017). Bactericidal activity of self-assembled palmitic and stearic fatty acid crystals on highly ordered pyrolytic graphite. Acta Biomater..

